# Sleep disturbance and work-related mental strain: A national prospective cohort study of the prediction of subsequent long-term sickness absence, disability pension and mortality

**DOI:** 10.1177/1403494820911813

**Published:** 2020-03-20

**Authors:** Torbjörn Åkerstedt, Ellenor Mittendorfer-Rutz, Syed Rahman

**Affiliations:** Department of Clinical Neuroscience, Karolinska Institutet, Sweden

**Keywords:** Work disability, stress, synergy, death

## Abstract

*Aims:* Sleep disturbances and work-related mental strain are linked to increased sickness absence and disability pension (DP), but we have no information on synergy effects. The aim of this study was to examine the combined (and separate) association of the two predictors with subsequent long-term work disability and mortality. *Methods:* A total of 45,498 participants aged 16–64 years were interviewed in the Swedish Surveys of Living Conditions between 1997 and 2013, and were followed up on long-term sickness absence (LTSA; >90 days/year), DP and mortality via national registers until 2016. Crude and multivariable Cox analyses were used to estimate hazard ratios (HR) and 95% confidence intervals (CI). *Results:* For LTSA, the HRs for sleep disturbances and work-related mental strain were 1.6 (95% CI 1.5–1.7) and 1.3 (95% CI 1.2–1.4), respectively. For DP, the HRs were 2.0 (95% CI 1.8–2.2) and 1.4 (95% CI 1.2–1.5). Mortality was only predicted by sleep disturbances (HR=1.2, 95% CI 1.1–1.4). No synergy effect was seen. ***Conclusions:* Work-related mental strain and, in particular, sleep disturbances were associated with a higher risk of subsequent LTSA and DP, but without synergy effects. Sleep disturbances were also associated with mortality. Exposure to interventions tackling sleep disturbance and prevention of workplace stress may reduce work disability.**

## Introduction

Stress is defined as physiological or mental activation/effort in response to demands [1]. Stress at work is often seen as a response to work demands or to work strain. The latter is quantified as the ratio between work demands and work control [2]. Work demands include items such as having to work fast, work hard, not having enough time or having to exert too much effort. This measure and particularly work strain are predictors of coronary heart disease [3,4]. The present study focuses on a related concept: work-related mental strain.

This concept has been used for more than 40 years in health surveys carried out by Statistics Sweden, but very little is known about its prospective association with health variables. However, there are indications that job strain predicts sickness absence (SA) [5–7]. The effect seems stronger in higher socio-economic groups [8]. Disability pension (DP) is also linked to prior job strain [9–11], but the studies are few. With regard to work demands as a predictor of all-cause mortality such a link has been demonstrated in several studies [12–14], whereas other studies have failed to find such a link [15–17]. No other studies seem to have focused on this outcome.

In his early work on stress, Selye emphasised that ‘recovery’ was a prerequisite for preventing a gradual increase in activation in response to stress [1]. Sleep is a major form of recovery, and sleep loss is frequently seen as a stressor [18]. Moreover, disturbed or short sleep is prospectively related to metabolic disease such as diabetes [19].

Sleep disturbances predict increased SA [20,21] and DP [22–25]. Sleep disturbances do not, however, seem to be related to mortality according to a recent meta-analysis [26].

Work-related mental strain and sleep disturbances are also prospectively linked to each other in a reciprocal way [27–28]. While work-related mental stress may increase activation, resulting in disturbed sleep, the latter might reduce the capacity to handle demands and thus result in more strain. This raises the issue of the combined effects of disturbed sleep on work-related mental strain on LTSA, DP and mortality. We hypothesised that there may be synergy effects and that there is a possibility that good sleep buffers against the negative effects of stress.

The purpose of the present study was to investigate the separate and combined associations of sleep disturbances and work-related mental strain with subsequent long-term sickness absence (LTSA), DP and mortality. To the best of our knowledge, no similar study has been carried out before.

## Methods

### Study population

A cohort of 62,575 individuals aged 16–64 years at baseline who participated in the Swedish Surveys of Living Conditions between 1997 and 2013 were identified. Face-to-face interviews were conducted between 1997 and 2005, and telephone interviews between 2006 and 2013. After exclusion of non-responders to the questions on sleep and work-related mental strain (*n*=17,077), the study population included 45,498 individuals. In addition, those who died or emigrated (*n*=18) between the interview and the start of follow-up were also excluded, leaving a sample size of 45,480 participants. Further, a subsample that excluded persons on disability or age-related pension at baseline (*n*=2916) was created for analyses with LTSA (defined as >90 net days of SA in one spell) or DP as the outcome. This research was carried out in accordance with the Declaration of Helsinki, and the study was approved by the Stockholm Regional Ethical Committee.

### Data sources

Individual-level information on this cohort was linked by using the unique personal (de-identified) identification number from: (a) the national patient register and cause of death register held by the National Board of Health and Welfare (date and cause/diagnosis of inpatient and specialised outpatient care and date and cause of death, respectively); (b) the Micro Data for Analysis of the Social Insurance database held by the Social Insurance Agency which includes information on date, grade and diagnoses of SA and DP; and (c) the Longitudinal Integration Database for Health Insurance and Labour Market Studies held by Statistics Sweden which includes data on age, sex, country of birth, education level, family situation and type of living area.

### Exposure measures

For exposure to sleep disturbances, individuals were asked: ‘Have you had trouble sleeping during the past two weeks?’ For work-related mental strain, the question was formulated as: ‘Is your work mentally straining?’ Note that the latter question was not time limited. They were measured once during the interview between 1997 and 2013. Up to 2007, the response options were only ‘yes/no’, but from 2008 onwards, participants could also answer ‘don’t know’ or ‘don’t want to answer’. Both the latter categories were categorised as non-response and were not included in any analyses.

### Outcome measures

Work disability as an outcome was measured as (a) LTSA and (b) granting of DP. Measurement of LTSA and DP were performed separately. Net days were calculated based on the grade (full time and part time) and duration of SA during follow-up. Mortality as an outcome was considered as all-cause mortality during follow-up. The outcome variables were categorised as yes/no.

### Covariates

Covariates (see categorisations in Table I) included sociodemographic measures (sex, age, educational level, type of living area, country of birth, family situation), health-related factors (specialised health-care use, body mass index (kg/m^2^), smoking), work-related factors (unemployment, job profile (2011–2013)) previous SA and interview method (with the categories telephone and face-to-face), and were measured at baseline.

**Table I.** Characteristics of the participants of the main cohort of individuals with disturbed sleep, work-related mental strain and fatigue between 1997 and 2013 in Sweden (*N*=45,498).

	*n*/%	No disturbed sleep or strain, *N*=21,512	Both disturbed sleep and strain, *N*=5385	*p*
Sex				
Male	22,986/50.5	12,188/56.7	2128/39.5	<0.0001
Female	22,512/49.5	9324/43.3	3257/60.5	
Age				
16–24	5226/11.5	2876/13.4	472/8.7	<0.0001
25–34	10,162/22.3	4750/22.1	1127/20.9	
35–44	11,548/25.4	5233/24.3	1398/26.0	
45–54	11,163/24.5	5082/23.6	1458/27.1	
5564	7426/16.3	3571/16.6	930/17.3	
Education level				
0–9 years	7243/15.9	4142/19.3	661/12.3	<0.0001
10–12 years	22,420/49.3	11,324/52.6	2410/44.8	
>12 years	15,835/34.8	6046/28.1	2314/42.9	
Type of living area^a^				
Large cities	16,338/35.9	7352/34.2	2174/40.4	<0.0001
Medium cities	16,198/35.6	7765/36.1	1836/34.1	
Rural areas	12,962/28.5	6395/29.7	1375/25.5	
Country of birth				
Sweden	40,614/89.3	19,415/90.3	4624/85.9	<0.0001
Other Nordic countries	1391/3.0	619/2.9	205/3.8	
Rest of the EU-25	905/2.0	364/1.7	133/2.5	
Rest of the world	2588/5.7	1114/5.2	423/2.5	
Family situation^b^				
Married/cohabiting, no children	6706/14.7	3216/14.9	863/16.0	0.0053
Single, no children	19,009/41.8	8852/41.2	2147/39.9	
Married/cohabiting, with children	14,998/33.0	7138/33.2	18,077/33.6	
Single, with children	2931/6.4	1169/5.4	442/8.2	
Living with parents <20 years	1795/4.0	1090/5.1	124/2.3	
Missing	59/0.1	47/0.2	2/0.0	
Specialised health-care use				
Non-user	14,287/31.4	6800/31.6	1747/32.4	<0.0001
Before start of follow-up	11,425/25.1	5125/23.8	1543/28.7	
During the follow-up	19,786/43.5	9587/44.6	2095/38.9	
BMI				
Under weight	634/1.4	288/1.3	103/1.9	0.0259
Normal weight	24,799/54.5	11,762/54.7	2893/53.7	
Overweight	15,471/34.0	7431/34.5	1798/33.4	
Obesity grade I	3365/7.4	1519/7.1	423/7.9	
Obesity grade II	551/1.2	250/1.1	68/1.3	
Obesity grade III	113/0.3	36/0.2	25/0.5	
Missing	565/1.2	226/1.1	75/0.5	
Daily smoker				
No	37,877/83.2	18,127/84.3	4330/80.4	<0.0001
Yes	7621/16.8	3385/15.7	1054/19.6	
LTSA				
No LTSA	39,226/86.2	19,086/88.7	4267/79.2	<0.0001
Previous LTSA	790/1.7	277/1.3	156/2.9	
Ongoing LTSA	713/1.6	199/0.9	199/3.7	
LTSA during follow-up	4769/10.5	1950/9.1	763/14.2	
DP				
No DP	41,794/91.8	20,185/93.8	4622/85.8	<0.0001
Ongoing DP	1494/3.3	518/2.4	317/5.9	
DP during follow-up	2210/4.9	809/3.8	446/8.3	
Unemployment				
None	41,007/90.3	19,132/89.1	4906/91.1	<0.0001
1–180 days	3715/8.1	1925/9.0	408/7.6	
>180 days	717/1.6	408/1.9	69/1.3	
Death during follow-up^c^	1701/3.7	825/3.8	221/4.1	0.3613
General anxiety/stress (yes)	7524/16.6	1866/8.68	2217/41.3	
Sleep disturbance	9849/21.7			
Work-related mental strain	19,513/42.9			
Fatigue (yes)	10,912/24.0	6360/29.6	3675/68.2	
Job profile^d^				
White collar	18,022/56.8	7072/49.0	2631/66.4	<0.0001
Blue collar	6370/20.1	3621/25.1	529/13.4	
Missing	7359/20.1	3741/25.9	801/20.2	

a Type of living area: big cities (Stockholm, Gothenburg and Malmö), medium-sized cities (cities with >90,000 inhabitants within 30 km distance from the centre of the city) and small cities/villages.

b Single=divorced/separated/widowed living with/without children.

c 12 died before follow-up.

d Available since 2001.

BMI: body mass index; LTSA: long-term sickness absence; DP: disability pension.

### Statistical analysis

Follow-up of participants began on 1 January following the interview year (between 1997 and 2013) and ended on 31 December 2016. Hazard ratios (HR) with 95% confidence intervals (CI) for mortality, LTSA and DP among the study population were estimated by Cox regression models after testing that the proportional hazard assumption was met. Individuals were followed until the date of LTSA, DP, death, emigration or the end of follow-up. Censoring was applied for death and emigration in case of an outcome other than death. Additionally, in the analyses for LTSA and DP as outcome, observations after turning 65 years of age (previous the mandatory retirement age) were censored. Moreover, in the analysis for LTSA as the outcome measure, censoring was applied for DP if granted during follow-up.

In order to identify the synergistic effects between sleep disturbance and work-related mental strain with regard to subsequent LTSA, DP and mortality, the additive interaction between sleep disturbance and work-related mental strain at baseline with regard to subsequent LTSA, DP and mortality was measured by calculating the relative excess risk due to interaction (RERI) and the synergy index (S) [29].

## Results

The number of LTSAs during follow-up was 4769, and the number of DPs was 2210. Among the respondents, 21.6% reported sleep disturbances, and 42.9% were experiencing mental strain related to work (Table I). Around 90% of the population did not have previous or ongoing work disability (i.e. LTSA or DP). During the follow-up period, 3.7% of the participants died, and 10.5% and 4.9% experienced LTSA and DP, respectively.

A descriptive analysis of the excluded survey participants who did not respond or who responded ‘don’t know’ to the questions related to the exposure measures (data not shown) revealed that they were predominantly male (62% vs. 50.5% among the responders), younger (16–24 years: 26% vs. 20%) and with a lower educational level (0–9 years: 43% vs. 21%), and a substantially higher proportion belonged to the ‘rest of the world’ in relation to country of birth (24% vs. 8%). In parallel, a higher proportion of the non-responders died during follow-up (6% vs. 3.7%), a higher proportion of them were treated by specialised health-care provider before the start of follow-up (31% vs. 25.1%) and nearly one third of such non-responders were already on DP at inclusion (32% vs. 3.3%). In contrast, a lower proportion of the non-responders were on LTSA during follow-up compared to responders (4% vs. 10.5%, respectively).

An association was observed between both exposure measures under study and LTSA in crude and adjusted models, but with higher HRs for sleep disturbances. We also observed similar associations with DP; the fully adjusted association for sleep disturbance with DP was stronger than that between work-related mental strain and DP (Table II). For mortality, only sleep had a significant albeit modest association with mortality (Table II). In sex-stratified analyses, the results were very similar in women and men (results not shown).

**Table II.** Hazard ratios (HR) and 95% Confidence Intervals (CI) for long-term sickness absence (LTSA), disability pension (DP) and mortality. Individuals reporting sleep disturbances, or work-related mental strain.

	Sleep disturbances			Work-related mental strain		
	*n* (%)	Model 1^a^	Model 2^b^	*n* (%)	Model 1^a^	Model 2^b^
LTSA (*N*=42,564)						
No	33,745 (79.3)	1	1	24,229 (56.9)	1	1
Yes	8819 (20.7)	**1.7 (1.6–1.8)**	**1.6 (1.5–1.7)**	18,335 (43.1)	**1.3 (1.2–1.3)**	**1.3 (1.2-1.4)**
DP (*N*=42,564)						
No	33,745 (79.3)	1	1	24,229 (56.9)	1	1
Yes	8819 (20.7)	**2.1 (1.9–2.3)**	**2.0 (1.8–2.2)**	18,335 (43.1)	**1.3 (1.2–1.4)**	**1.3 (1.2–1.5)**
Mortality (*N*=45,480)						
No	35637 (78.4)	1	1	25,970 (57.1)	1	1
Yes	9843 (21.6)	**1.2 (1.1–1.4)**	**1.2 (1.1–1.4)**	19,510 (42.9)	0.9 (0.8–1.0)	1.0 (0.9–1.1)

Statistically significant values (*p*<0.05) are shown in bold. Individuals with ongoing DP (*n*=1235) or age-related pension (*n*=1423) or both (*n*=258) at start were excluded.

a Crude model.

b Adjusted for sex, age, educational level, type of living area, family situation, country of birth, previous health-care use, previous sickness absence, smoking status, BMI and unemployment benefit.

In a sensitivity analysis, we also adjusted for work-related mental strain in the analysis of sleep disturbances, and vice versa. This did not change any of the estimates appreciably other than a minor reduction of the HR for LTSA and DP (not shown). For mortality, the HR for sleep disturbance was no longer significant when adjusted for work-related mental strain (HR=1.0, 95% CI=0.9–1.1).

Synergistic effects between sleep disturbance and mental strain on LTSA, DP or mortality were not found. The RERI statistic was between 0.0 and 0.2, and the S statistic varied between 1 and 1.2 across the models, neither of which indicated statistical significance (data not shown). However, individuals experiencing both sleep disturbance and mental strain were at a significantly higher risk for LTSA and DP (Table III) than those who reported only one of the exposures (compared to those who did not report any such exposures). Post-hoc power analyses (α=0.05 and 0.01) indicated 100% power for all three analyses of dependent variables.

**Table III.** Synergistic effect of sleep disturbances and work-related mental strain in relation to LTSA, DP and mortality.

Sleep disturbance	Work-related mental strain	*n* (%)	Model 1^a^	Model^b^
*LTSA*				
−	−	20,303 (47.7)	1	1
+	−	3926 (9.2)	**1.7 (1.6–1.9)**	**1.6 (1.5–1.7)**
−	+	13,442 (31.6)	**1.2 (1.1–1.3)**	**1.3 (1.2–1.3)**
+	+	4893 (11.5)	**2.0 (1.8–2.1)**	**1.9 (1.8–2.1)**
*DP*				
−	−	20,303 (47.7)	1	1
+	−	3926 (9.2)	**2.1 (1.8–2.4)**	**2.0 (1.7–2.3)**
−	+	13,442 (31.6)	**1.2 (1.1–1.3)**	**1.3 (1.1–1.4)**
+	+	4893 (11.5)	**2.3 (2.1–2.6)**	**2.4 (2.2–2.7)**
*Mortality*				
−	−	21,521 (47.3)	1	1
+	−	4458 (9.8)	**1.3 (1.1–1.5)**	1.2 (1.0–1.4)
−	+	14,125 (31.1)	0.8 (0.8–0.9)	1.0 (0.9–1.1)
+	+	5385 (11.8)	1.2 (1.0–1.4)	**1.2 (1.1–1.4)**

Individuals with ongoing DP (*n*=1235) or age-related pension (*n*=1423) or both (*n*=258) at start were excluded. Statistically significant values (*p*<0.05) are shown in bold.

a Crude model.

b Adjusted for sex, age, educational level, type of living area, family situation, country of birth, previous health-care use, previous sickness absence, smoking status, BMI and unemployment benefit.

## Discussion

Sleep disturbances and work-related mental strain were significantly associated with higher risk of subsequent LTSA and DP. Mortality was weakly associated with sleep disturbances. No synergy effects were seen, but the two predictors combined yielded higher HRs for LTSA and DP than for either of them separately.

The absence of synergy was unexpected in view of the clear associations with LTSA and DP of the two predictors separately. It may be the case that the association between sleep disturbances and work-related mental strain is too high to permit independent contributions. Still, the HR of the combined exposure to the two variables was higher than that of either of them alone. This should be followed up. Possibly, the effects might have been larger if the exposure had not been measured through a yes/no response alternative. More graded responses may have made possible a more optimal cut-off point with higher mental strain. This should be followed up in future studies.

The significant association between sleep disturbances and long-term SA [20–21] and DP [22–25] confirms previous work. One possible explanation for these findings may be that subjective health is reduced after disturbed sleep as it is after experimental sleep loss [30]. It should be noted that the sleep disturbances in the present study referred to the preceding two weeks only. A longer reference span may have yielded stronger results.

The significant association between work-related mental strain and LTSA agrees with previous work with job strain as a predictor [5–7]. The present results on mental work strain and DP are also in line with studies of job strain and DP [9–11]. Work stress also shows similar links with DP [31]. A possible explanation for these findings may be the lower level of subjective health in individuals under high levels of work strain [32].

For both LTSA and DP, the HR associated with sleep disturbances was higher than for work-related mental strain. This suggests that disturbed sleep may be more important with respect to these measures of work disability. The former referred to the two recent weeks, while mental work strain did not have a temporal reference. This could have led to attenuation of the association of mental work strain. Still, it is quite clear that reporting sleep disturbances during the recent two weeks is a predictor of work disability.

The lack of association between work-related mental strain and mortality agrees with some previous studies [15–17], but disagrees with others that found an association [12–14]. Apparently, it is not possible to draw any firm conclusions on the association between work-related mental strain and mortality. It might be that exposure to work-related mental strain shows a strong variation across time and that baseline exposure may not be representative for the accumulation across follow-up time. At present, there does not seem to be any previous work on such variation over time, apart from the observation that job demands decrease across eight years of aging in both blue- and white-collar workers [33]. Such effects may also have been involved in the present study.

The lack of association between disturbed sleep and mortality in the present study (after adjusting for mental work strain) agrees with a recent meta-analysis [26]. This observation contrasts with what would be expected from the links between sleep disturbances and SA and DP in the present study, as well as with similar previous work [22–25]. It also contrasts with the observations of both long and short sleep duration being associated with increased mortality risk [34], even if poor sleep does not necessarily imply short sleep. It is also well established that sleep quality and duration vary over time [35], and, as with work-related mental strain, it is possible that baseline measures may not be representative of exposure to that predictor during follow-up.

Some of the limitations of the present study have been discussed above. One additional limitation is that cross-sectional measures of the exposure can create possibilities for reverse causality between the exposure and the outcome. For this purpose, we controlled our analyses for previous SA and use of specialised health care. Another limitation is that exposure was determined through self-report, but this may be difficult to avoid when focusing on the present types of exposure in a large sample. Furthermore, the exposure measurements were based on surveys, and participants are likely to be healthier than the general population. About 21% of the sample did not respond to the questions on exposure and covariates. A description of this group from the registers indicated that they showed more disease and more DP, and originated from other parts of the world than Sweden. This may have attenuated the links between exposures and outcomes.

The strengths of this study include its base in data from high-quality Swedish population-based nationwide registers with longitudinal data linked at the individual level. The large sample size of more than 40,000 offers satisfactory statistical power and also represents a strength of the study. Additionally, information regarding the covariates and outcome were derived from nationwide registers. The use of register data eliminated the possibility of recall bias regarding outcome measures and avoided the loss to follow-up. Additionally, a wide range of socio-demographic, medical and work-related factors could be included as potential confounders.

In conclusion, work-related mental strain and, in particular, sleep disturbances were associated with a higher risk of subsequent LTSA and DP, but no synergy effects were present. Sleep disturbances were also significantly related to mortality. Exposure to interventions tackling sleep disturbance and prevention of workplace stress may be key to reducing work disability. The conclusions should probably not be generalised to groups in poor health at the start or to immigrant groups.
